# Overexpression of CARMA3 in Non-Small-Cell Lung Cancer Is Linked for Tumor Progression

**DOI:** 10.1371/journal.pone.0036903

**Published:** 2012-05-15

**Authors:** Zixuan Li, Lianyue Qu, Qianze Dong, Bo Huang, Haiying Li, Zhongping Tang, Ying Xu, Wenting Luo, Lifeng Liu, Xueshan Qiu, Enhua Wang

**Affiliations:** 1 Department of Pathology, First Affiliated Hospital and College of Basic Medical Sciences, China Medical University, Shenyang, People’s Republic of China; 2 Department of Pharmacy, First Affiliated Hospital of China Medical University, Shenyang, People’s Republic of China; 3 Department of Pathology, Liaoning Cancer Hospital, Shenyang, People’s Republic of China; 4 Department of Orthopaedics, First Affiliated Hospital of China Medical University, Shenyang, People’s Republic of China; Queen Elizabeth Hospital, Hong Kong

## Abstract

We aimed to investigate the clinical significance of the expression of novel scaffold protein CARMA3 in non-small-cell lung cancer (NSCLC) and the biological function of CARMA3 in NSCLC cell lines. We observed moderate to high CARMA3 staining in 68.8% of 141 NSCLC specimens compared to corresponding normal tissues. The overexpression of CARMA3 was significantly correlated with TNM stage (P = 0.022) and tumor status (P = 0.013). CARMA3 upregulation also correlated with a shorter survival rate of patients of nodal status N0 (P = 0.042)as well as the expression of epidermal growth factor receptor (EGFR) (P = 0.009). In EGFR mutation positive cases, CARMA3 expression was much higher (87.5%) compared to non-mutation cases (66.1%). In addition, we observed that knockdown of CARMA3 inhibits tumor cell proliferation and invasion, and induces cell cycle arrest at the boundary between the G1 and S phase. We further demonstrated a direct link between CARMA3 and NF-κB activation. The change of biological behavior in CARMA3 knockdown cells may be NF-κB-related. Our findings demonstrated, for the first time, that CARMA3 was overexpressed in NSCLC and correlated with lung cancer progression, EGFR expression, and EGFR mutation. CARMA3 could serve as a potential companion drug target, along with NF-kB and EGFR in EGFR-mutant lung cancers.

## Introduction

CARMA3 (also known as CARD10 or Bimp1) belongs to the CARMA family and contains three members, CARMA1 (also known as CARD11), CARMA2 (CARD14), and CARMA3. These three proteins share similar structural motifs, contain a CARD domain, a Src-homology 3 (SH3) domain, one or several PDZ domains, and a GuK domain. However, they exhibit distinct expression profiles; CARMA1 is expressed in hematopoietic cells, CARMA2 in the placenta, and CARMA3 in all non-hematopoietic cells [1]–[4]. Recent studies have demonstrated that CARMA3, as a scaffold protein, plays a critical role in GPCR ligands and PKC-induced NF-κB activation [5]–[8]. It has been previously demonstrated that NF-κB activation is involved in tumorigenesis and in the development of neural, heart, and immune diseases [9]–[13]. CARMA3 activates NF-κB by recruiting Bcl10 and MALT1, two indispensable proteins that are required for GPCR and PKC-induced NF-κB activation [6], [14]–[16]. The PKCa-CARMA3 signaling axis plays an essential role in LPA-induced ovarian cancer cell in vitro invasion [17]. A recent study demonstrated that CARMA3 deficiency impaired cancer cell proliferation in vitro and in vivo, and inhibited survival, migration, and invasion in human breast cancer cell lines MDA468 and A431 [18]. CARMA3 knockdown caused marked inhibition of SDF-1α mediated invasion of oral squamous cell carcinoma TB2–T4 cells [7]. However, the protein expression of CARMA3 in lung cancer tissues and the potential role of CARMA3 in the biological behavior of lung cancer cells have not been explored. Thus, we examined the expression of CARMA3 in lung cancer tissues and its relationship with various clinicopathological parameters. In addition, we assessed the association of CARMA3 expression with the proliferation and metastatic potential of several NSCLC cell lines.

## Materials and Methods

### Patients and Specimens

This study was approved by the Ethics Committee of the China Medical University, and written informed consent was obtained from each patient. 141 cases of NSCLC samples were obtained form the First Affiliated Hospital of China Medical University during the period of 2008 to 2011. The histological diagnosis and grade of differentiation of the tumors were defined by evaluation of the hematoxylin and eosin-stained tissue sections, according to the World Health Organization guidelines of classification. All 141 specimens were re-evaluated with respect to their histological subtypes, differentiation status, and tumor stages. For NSCLC samples, SCC and adenocarcinoma were identified in 65 and 76 of the 141 cases, respectively. Lymph node metastases were observed in 68 of the 141 patients. The p-TNM taging system of the International Union Against Cancer (7th Edition) was used to classify specimens as stages I (n = 64), II (n = 41), III (n = 30) and IV (n = 6).

### Cell Lines

A549,H1299,HBE and H460 were obtained from American Type Culture Collection (Manassas, VA, USA), LK2 was obtained from the Japanese Cancer Research Resources Bank (Tokyo,Japan),SPC were obtained from CCTCC (Chinese Center of Typical Culture Conserve, Wuhan, P.R.China), H157 was purchased from Cell Bank, Chinese Academy of Sciences (Shanghai, China), The BE1 and LH7 were gifts from Dr. Jie Zheng (College of Medicine, Beijing University, China).The cells were cultured in RPMI 1640 (Invitrogen, Carlsbad, CA, USA) containing 10% fetal calf serum (Invitrogen), 100 IU/ml penicillin (Sigma, St. Louis, MO, USA), and 100 µg/ml streptomycin (Sigma). Cells were grown on sterile tissue culture dishes and were passaged every 2 days using 0.25% trypsin (Invitrogen).

### Immunohistochemistry

Surgically excised tumor specimens were fixed with 10% neutral formalin, embedded in paraffin and 4 µm thick sections were prepared. Immunostaining was performed using the avidin–biotin–peroxidase complex method (Ultra Sensitive TM, Maixin, Fuzhou, China). The sections were deparaffinized in xylene, rehydrated in graded alcohol series and boiled in 0.01 M citrate buffer (pH 6.0) for 2 minutes in an autoclave. Endogenous peroxidase activity was blocked using hydrogen peroxide (0.3%), which was followed by incubation with normal goat serum to reduce non-specific binding. Tissue sections were incubated with CARMA3 rabbit polyclonal antibody (1∶80 dilution) (Sigma). Mouse immunoglobulin (at the same concentration of the antigen specific antibody) (Maixin, Fuzhou, China) was used as a negative control. Staining for all primary antibodies was performed at room temperature for 2 hours. HRP-Polymer Conjugated anti Mouse/Rabbit IgG (ready-to-use ) (Maixin, Fuzhou, China) was used as the secondary antibody. After washing, the sections were incubated with horseradish peroxidase-conjugated streptavidin–biotin, followed by 3, 3′-diaminobenzidine tetrahydrochloride to develop the peroxidase reaction. Counterstaining of the sections was done with hematoxylin, which were then dehydrated in ethanol before mounting.Two independent investigators examined all tumor slides randomly. Five views were examined per slide, and 100 cells were observed per view at 400×magnification. Normal bronchial epithelium present in the tumor sections was used as negative control. Immunostaining of CARMA3 was scored following a semi-quantitative scale by evaluating in representative tumor areas, the intensity and percentage of cells showing higher immunostaining than the control cells. cytoplasmic staining of the tumor cells was considered as positive immunostaining. The intensity of CARMA3 cytoplasmic staining was also scored as 0 (no staining), 1 (weak), 2 (marked). Percentage scores were assigned as 1- 1–25%, 2- 26–50%and 3- 51–100%. The scores of each tumor sample were multiplied to give a final score of 0 to 6 and the total expression of CARMA3 was determined as either negative or low expression (-): score <3 or positive expression or high expression (+): score ≥3.

**Figure 1:**
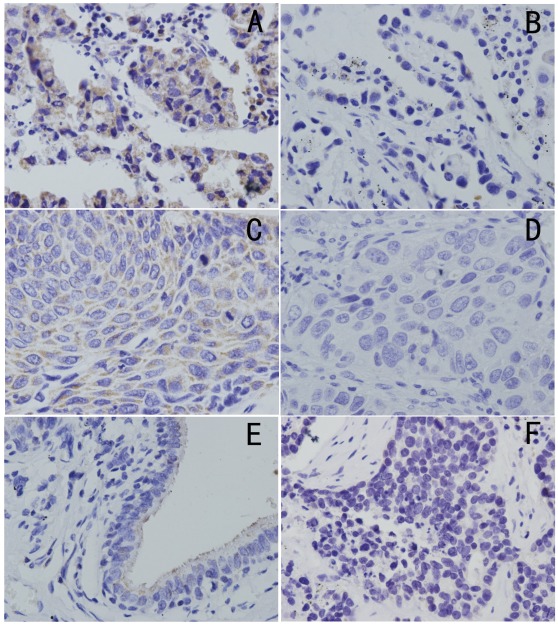
Immunohistochemical staining of CARMA3 in lung cancer tissue sections. (A) Strong CARMA3 expression in lung adenocarcinoma(400×). (B) Weak CARMA3 expression in lung adenocarcinoma(400×). (C) Strong CARMA3 expression in lung squamous cell carcinoma(400×). (D) Weak CARMA3 expression in lung squamous cell carcinoma(400×). (E) Normal bronchial epithelium showing weak staining for CARMA3(400×). (F) Negative controls for CARMA3 staining with non-immune mouse IgG antibody(400×).

**Table 1:** Distribution of CARMA3 status in NSCLC according to clinicopathological characteristics.

Characteristics	patients	CARMA3 positive	CARMA3 negative	*P*
Age				
<60	74	49(66.22%)	25(33.78%)	0.305
≥60	67	48(71.64%)	19(28.36%)	
Gender				
Male	83	53(63.86%)	30(36.14%)	0.091
Female	58	44(75.86%)	14(24.14%)	
Histology				
Adenocarcinoma	76	57(75.00%)	19(25.00%)	0.062
Squamous cell carcinoma	65	40(61.54%)	25(38.46%)	
Differentiation				
Well	63	43(68.25%)	20(31.75%)	0.522
Moderate- Poor	78	54(69.23%)	24(30.77%)	
TNM stage				
I	64	38(59.38%)	26(40.63%)	0.022
II+III+IV	77	59(76.62%)	18(23.38%)	
Tumor status				
T1–T2	75	45(60.00%)	3040.00%)	0.013
T3–T4	66	52(78.79%)	14(21.21%)	
Nodal status				
N0	73	46(63.01%)	27(36.99%)	0.088
N1 N2 N3	68	51(75.00%)	17(25.00%)	
EGFR status				
Negative	61	35(57.38%)	26(42.62%)	0.009
Positive	80	62(77.50%)	18(22.50%)	

**Figure 2:**
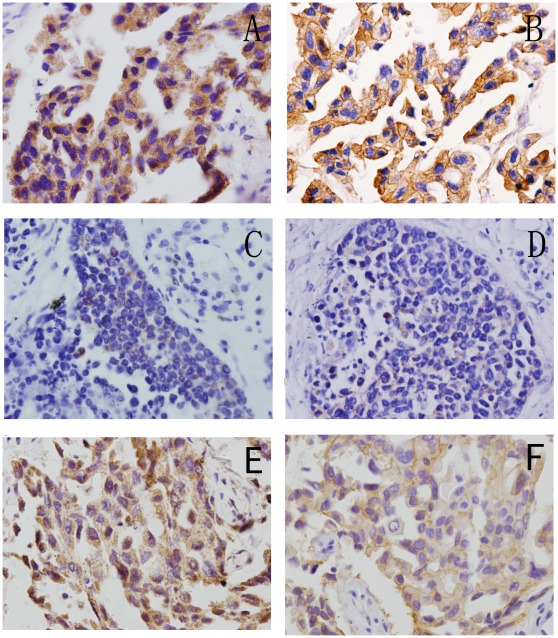
Examples of CAMRA3 and EGFR expression in NSCLC cases. Immunohistochemical staining for CARMA3 and EGFR in 2 representative NSCLC samples. One showed strong expression of CARMA3 (A) and strong EGFR expression (400×) (B). The other showed weak expression of CARMA3(400×) (C) and weak EGFR expression(400×) (D). (E,F) Correlation of CARMA3 and EGFR expression in NSCLC samples with EGFR mutation(400×).

### Analysis of EGFR Mutation

Paraffin sections of 75 NSCLC specimens for EGFR mutation analysis were obtained from First Affiliated Hospital of China Medical University between 2009 and 2011. Briefly, DNA was extracted from paraffin-embedded tissue sections using TaKaRa DEXPAT Easy Kit (TAKARA Biotechnology, Dalian, China) according to the manufacturer’s protocol. EGFR mutations were detected using EGFR mutation detection kit (GP Medical Technologies, Beijing, China) on 7900HT Fast Real-Time PCR System (Applied Biosystems,Foster City,Calif).

### Quantitative Real-time PCR (SYBR Green Method)

Quantitative real-time PCR was performed using SYBR Green PCR master mix (Applied Biosystems) in a total volume of 20 µl on 7900HT Fast Real-Time PCR System (Applied Biosystems) as follows: 95°C for 30 seconds, 40 cycles of 95°C for 5 seconds, 60°C for 30 seconds. A dissociation step was performed to generate a melting curve to confirm the specificity of the amplification. β-actin was used as the reference gene. The relative levels of gene expression were represented as ΔCt = Ct gene –Ct reference, and the fold change of gene expression was calculated by the 2-ΔΔCt method. The primer sequences are provided in Table S1, Experiments were repeated in triplicate.

### Western Blot Analysis

Total proteins from primary tissues and cell lines were extracted in lysis buffer (Thermo Fisher Scientific,Rockford,IL) and quantified using the Bradford method. Fifty micrograms of protein were separated by SDS–PAGE (12%). After transferring, the polyvinylidene fluoride (PVDF) membranes (Millipore, Billerica, MA, USA) were incubated overnight at 4°C with the following antibodies CARMA3(1∶200; Sigma), Bcl-10 and β-actin (1∶500; Santa Cruz Biotechnology, Santa Cruz,CA),anti-phospho-ERK, anti-phospho-IKK,anti-phospho-IκB, anti-phospho-Rb,anti-P27, anti-Cyclin A, anti-Cyclin D1, anti-Cyclin D3, anti-CDK4 and anti-CDK6(1∶1000; Cell Signaling Technology,Danvers,MA). After incubation with peroxidase-coupled anti-mouse or rabbit IgG (Santa Cruz Biotechnology) at 37°C for 2 hours, bound proteins were visualized using ECL (Thermo Fisher Scientific) and detected using BioImaging Systems (UVP Inc., Upland, CA, USA). The relative protein levels were calculated based on β-actin as the loading control.

### Small Interfering RNA Treatment

Dharmacon siGENOME SMARTpool siRNA for CARMA3,Bcl10 and Dharmacon siGENOME Non-targeting siRNA #1 were purchased from Dharmacon (ThermoFisher Scientific). For transfections, cells were seeded in a 24-well plate 24 h before the experiment. The cells were transfected with siRNA using the DharmaFECT 4 (0.20 µL/well; ThermoFisher Scientific) according to the manufacturer’s protocol. Following transfection, the mRNA and protein levels were assessed 48 hours later.

### Cell Proliferation Test

Cell proliferation assay was performed using Cell Counting Kit-8® solution (Dojindo, Gaithersburg, MD) according to the manufacturer’s protocol. Briefly, cells were seeded at a concentration of 5×10^3^ cells/100 µl/well in 96-well culture plates and treated with 10 µl/well of Cell Counting Kit-8® solution during the last 4 hours of the culture. Optical density of the wells was measured at 450 nm using a microplate reader.

### Colony Formation Assay

For colony formation assays, cells were grown on 6 cm dishes at a density of 5,000 cells/dish, and transfected with CARMA3, Bcl10, or negative control siRNAs. Colonies were washed twice with PBS, fixed, and stained with formaldehyde-crystal violet 13–15 days after transfection. All experiments were independently repeated a minimum of three times under identical conditions.

**Figure 3:**
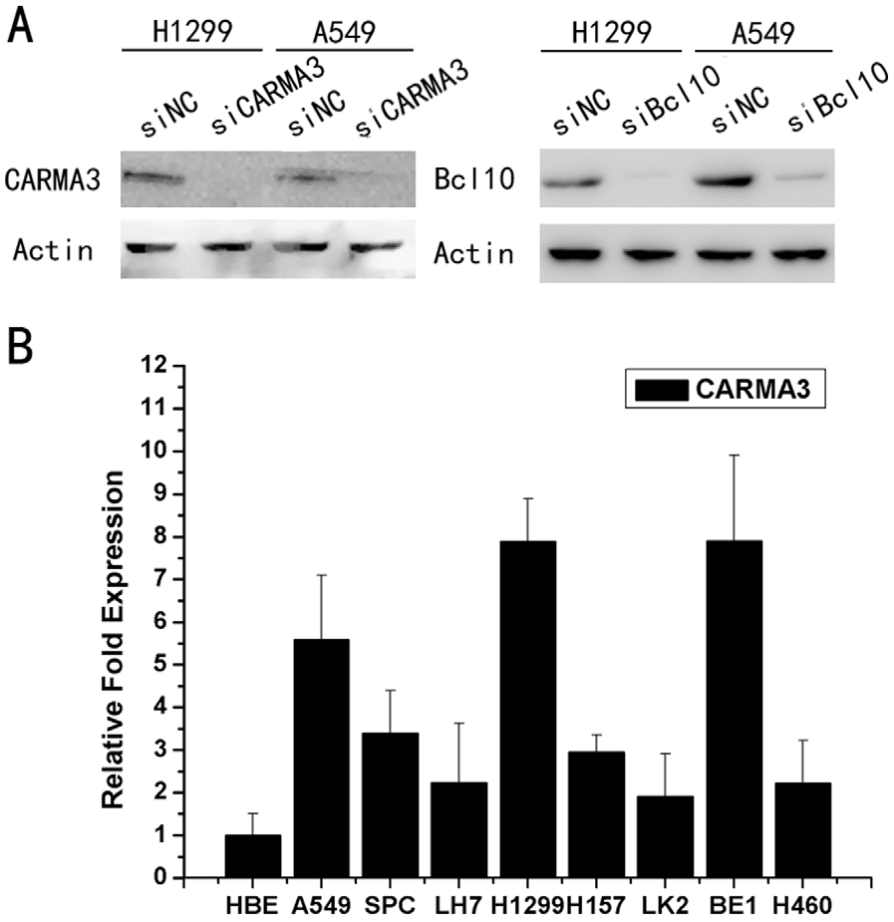
CARMA3 depletion in H1299 and A549 cells. (A) Western blot analysis of CARMA3 expression in lung cancer cells transfected with siRNA. (B) Relative CARMA3 expression profiles in a panel of human airway derived cell lines assessed by real-time PCR.

**Figure 4:**
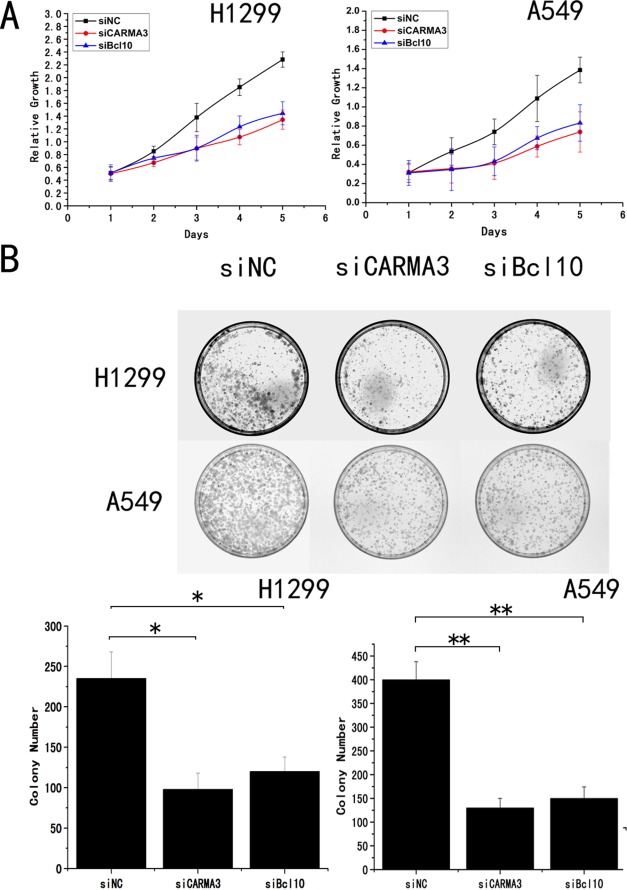
CARMA3 depletion inhibits cancer cell proliferation. (A) Cell growth rate was determined by the CCK-8 assay, as described in Section 2. The data are mean ± SD of three independent experiments. (B) (Upper panel) H1299 and A549 cells transduced with siRNA for control, CARMA3, or Bcl10 were subjected to colony formation assay. (lower panel) Histogram quantification. Error bars indicate ±SD (*p<0.05, **p<0.01).

**Figure 5:**
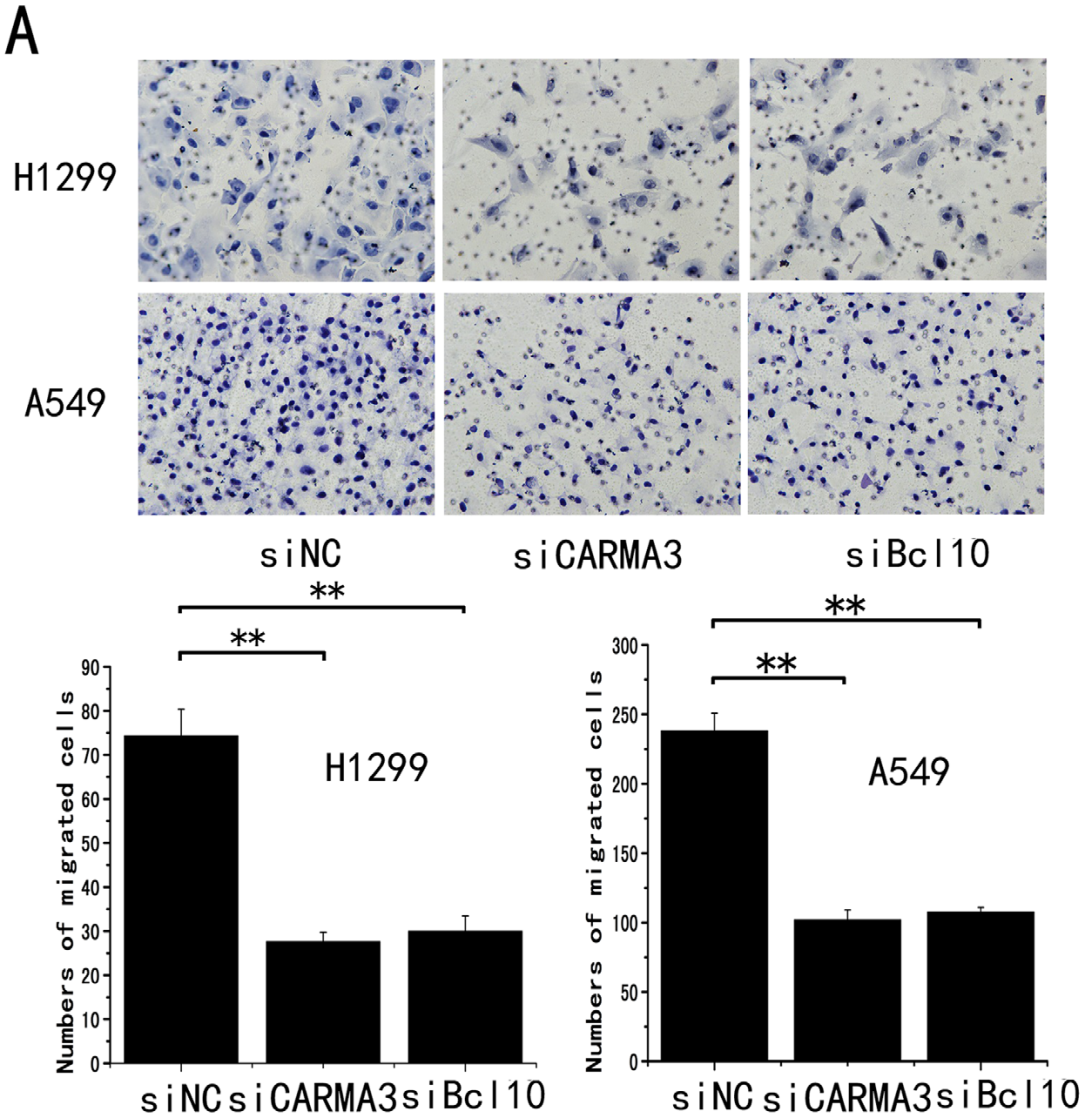
Knockdown of CARMA3 expression in H1299 and A549 cells inhibited cell invasion in a Boyden chamber assay. (Upper) CARMA3 and Bcl10 siRNA treatment inhibited measurable cell invasion in both cell lines(200×). (Lower) The bar graph indicates the mean and difference in cell number among the photographs, and the standard derivation was calculated from the average of cells in the 10 fields (**, P<0.01).

### Matrigel Invasion Assay

Cell invasion assay was performed using a 24-well Transwell chamber with a pore size of 8 µm (Costar,Cambridge,MA). The inserts were coated with 20 µl Matrigel (1∶3 dilution, BD Bioscience, San Jose, CA,USA). Forty-eight hours after the transfection, cells were trypsinized and 3×10^5^ cells in 100 µl of serum-free medium were transferred to the upper Matrigel chamber and incubated for 16 hours. After incubation, the non-invaded cells on the upper membrane surface were removed with a cotton tip, and the cells that passed through the filter were fixed with 4% paraformaldehyde and stained with hematoxylin. The number of invaded cells was counted in 10 randomly selected high power fields under the microscope. This experiment was performed in triplicate.

### Cell Cycle Analysis

Cells (500,000) were seeded into 6-cm tissue culture dishes. After 12 h of incubation, cells were transfected with indicated amounts of siRNA. Cells were fixed with 75% ethanol 48 h after transfection, washed with phosphate-buffered saline (PBS), and stained in 5 mg/ml propidium iodide in PBS supplemented with RNase A (Roche, Indianapolis, IN) for 30 minutes at room temperature. Data were collected using BD systems.

### Luciferase Assay

Cells were initially transfected with CARMA3, Bcl10, or negative control siRNAs for 24 h; cells were then co-transfected with 500 ng pNF-kB-Luc reporter plasmid, and 100 ng Renilla luciferase plasmid for 24 h. The cells were treated for 30 min with or without EGF (100 ng/ml), and then lysed; luciferase activity was determined with a dual-luciferase reporter assay system (Promega, Madison, WI, USA) according to the manufacturer’s instructions. NF-kB activity was assessed upon normalization of firefly luciferase activity to Renilla luciferase activity. Experiments were performed in triplicate in three independent experiments.

### Statistical Analysis

SPSS version 16.0 for Windows was used for all analyses. The Chi-squared test was used to examine possible correlations between CARMA3 expression and clinicopathologic factors. Kaplan–Meier curves were used for survival analysis, and log-rank was determined based on the differences. The Student’s t-test was used to compare other data. p-values were based on the two-sided statistical analysis, and p<0.05 was considered to indicate statistical significance.

**Figure 6:**
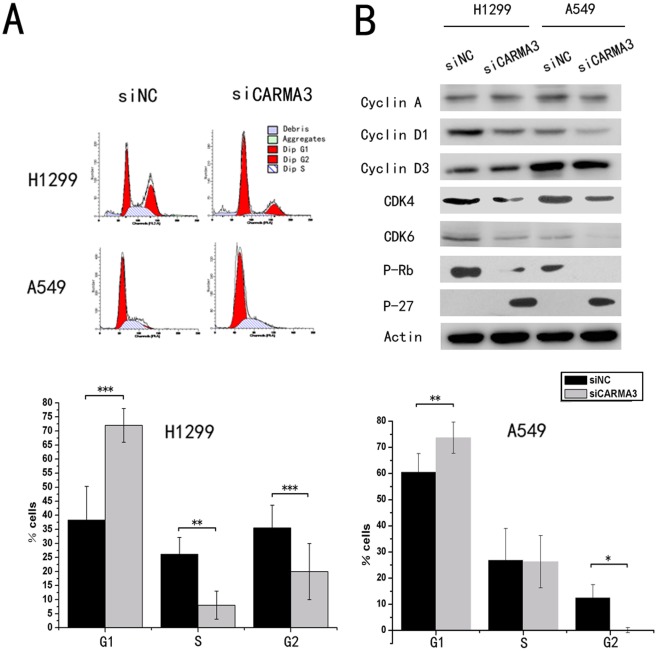
Cell cycle distribution analysis. (A) (Upper) H1299 and A549 cells were treated with CARMA3-specific siRNA for 48 h. Next, the cells were harvested and treated with RNase, and stained with PI. The cell cycle distribution was analyzed by flow cytometry. (Lower) The percentage of cells in G1,S,G2 phase in histograms. (*p<0.05, **p<0.01, ***p<0.001) versus control group. The results are representative of at least three independent experiments. (B) H1299 and A549 cells were treated with CARMA3-specific siRNA for 48 h. Cell lysates were subjected to immunoblotting analysis using indicated antibodies.

**Figure 7:**
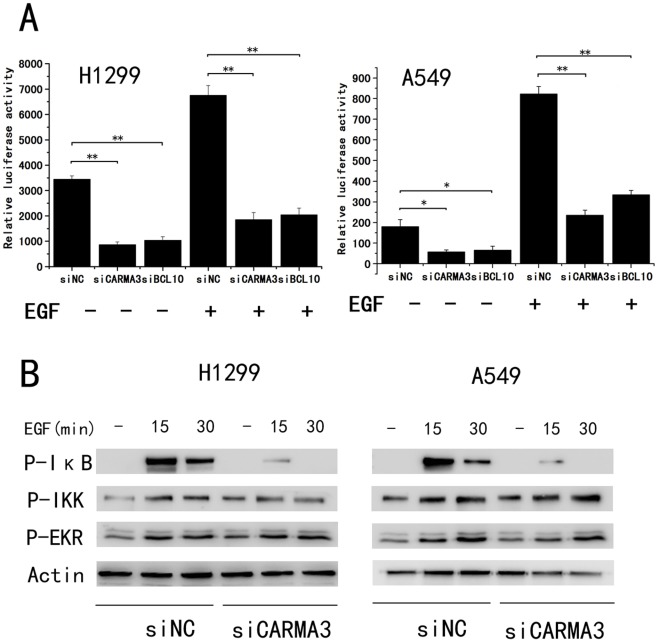
CARMA3 is required for NF-κB activation in NSCLC. (A) H1299 and A549 cells were transfected with control or CARMA3-specific siRNA for 24 h, then treated with NF-κB-luciferase and control *Renilla* reporter plasmids for 24 h. The cells were treated with or without EGF (100 ng/ml) before harvest, and the resulting NF-κB induction was measured by calculating the luciferase/*Renilla* ratio. Data (mean_S.E.) are from at least three separate determinations. (**p<0.01) (B) H1299 and A549 cells were transfected with an siRNA pool targeting CARMA3 for 48 h, then treated with EGF (100 ng/ml) for the indicated periods of time. Total cell lysates were prepared from these cells and then subjected to immunoblotting analysis with indicated antibodies.

## Results

### Clinical Significance of CARMA3 Protein Expression in NSCLC Tissues

We analyzed the protein expression levels of CARMA3 using immunohistochemistry of 91 NSCLC specimens and their corresponding normal tissues. CARMA3 was found to be predominately expressed in the cytoplasmic compartment. Normal bronchial epithelia exhibited negative or weak CARMA3 staining (Fig. 1E), whereas CARMA3 staining was significantly stronger in lung cancer tissues. We found moderate to high CARMA3 staining in 69% of the lung cancer specimens (Fig. 1A and C). We also use western-blotting to analyze the expression of CARMA3 in 18 cases.The results show that 56% of the lung cancer specimens show moderate to high CARMA3 expression (Figure S2, S3).

The correlation between CARMA3 expression and the clinicopathologicfactors of NSCLC is shown in Table 1. CARMA3 overexpression exhibited a significant correlation with TNM stage (P = 0.022) and tumor status (P = 0.013), but no correlation was observed concerning age, sex, differentiation, lymph node metastases, and tumor histology. To better claim the correlation of CARMA3 with lung cancer progression, we evaluated the relationship between the expression of CARMA3 and overall survival of N0 stage NSCLC patients(38 CAMRA3+ and 16 CAMRA3 - ). After Kaplan-Meier survival analysis, we found that patients with low expression of CARMA3 had a statistically significant longer survival(median survival = 32±3.80 months; 95% confidence interval 24.56–39.44 months) than those with high expression of CARMA3(median survival = 20±3.91months; 95% confidence interval 12.33–27.67 months; P = 0.042; Figure S4).

A recently reported study demonstrated that CARMA3 expression is required for EGFR induced NF-κB activation [15]. EGFR overexpression has been demonstrated in many human cancers, including lung, colon, pancreas, breast, ovary, bladder, kidney, and the nervous system [19], [20]. Thus, we examined the relationship between CARMA3 expression and EGFR status in NSCLC. We found that EGFR was expressed in the cell membrane and cytoplasmic. A significant correlation between CARMA3 expression and expression of EGFR was observed (P = 0.009) in the NSCLC samples (Table 1 and Fig. 2A–D). To determine whether the EGFR mutation is correlated with the elevated CARMA3 expression, we investigated the relationship between CARMA3 expression and EGFR mutation. We used an EGFR mutation detection kit, and found 16 of 75 tumor samples were positive for EGFR mutation (Table S2). Among the 16 cases, 87.5% exhibited positive CARMA3 expression, a much higher rate compared to non-mutation cases (66.1%). We found that 86.67% EGFR positive mutation cases also exhibited positive CARMA3 expression (Fig. 2E and F).

### CARMA3 Contributes to Lung Cancer Cell Growth and Invasion

We first examined the expression of CARMA3 in several lung cancer cell lines, as shown in Fig. 3B, H1299, A549, BE1 exhibited high expression levels of CARMA3 whereas normal bronchial epithelium (HBE) cells, LK2, and LH7 exhibited lower levels of CARMA3 expression. SPC, H157, and H460 exhibited moderate levels of CARMA3 expression.

We next employed an RNA interference approach to determine whether CARMA3 and CBM complex (CARMA3-Bcl10-MALT1) plays a role in cancer cell growth. The CARMA3 and Bcl10 expression levels were unchanged upon transient transfection with the negative control siRNA, whereas CARMA3- or Bcl10-specific siRNA significantly suppressed both mRNA as well as protein expression levels in the H1299 and A549 cell lines (Fig. 3A and Fig. S1). Next, we examined the cell growth rate. With CARMA3 or Bcl10 siRNA, H1299 cells displayed reduced growth rates compared to the negative control (Fig. 4A). Similar to H1299 cells, we found that knockdown of CARMA3 or Bcl10 also reduced the growth rate of A549 cells (Fig. 4A). We utilized an independent method, colony formation assays, to validate the anti-proliferative effects of CARMA3 or Bcl10 inhibition in lung cancer cells. CARMA3- or Bcl10-targeting siRNAs led to a clear reduction of the colony formation capacity of two tested lung cancer cell lines compared to control siRNA-treated cells (Fig. 4B). These loss-of-function studies demonstrated that the CARMA3 and CBM siRNAs could inhibit tumor cell proliferation compared to irrelevant siNC. To further examine whether CARMA3 and Bcl10 contribute to the invasive capabilities of non-small cell lung cancer cells, we conducted matrigel invasion assays. Our results demonstrated that the invasive capabilities of CARMA3- and Bcl10-knockdown H1299 cancer cells were reduced compared to the negative control cells (Fig. 5). Similarly, the knockdown of CARMA3 or Bcl10 in A549 cells also reduced the invasive capabilities of these cells (Fig. 5). Taken together, these results demonstrate that CARMA3 and CBM complex are positive regulators cell invasion.

### CARMA3 Deletion Results in G1 Arrest and Growth Inhibition of Lung Cancer Cells

Cell cycle analyses by fluorescence activated cell sorting (FACS) were performed in cancer cells with or without CARMA3-knockdown, and we found that the percentage of cells in the G1 phase was increased and cells in the S phase was decreased in cells with CARMA3-knockdown compared to control cells (Fig.6 A). These results suggest that CARMA3 deletion induces cell cycle arrest at the G1/S boundary. To investigate the mechanism underlying the induction of the cell cycle arrest, we tested the effect of CARMA3-knockdown on Cyclin A, Cyclin D1, Cyclin D3, CDK4, CDK6, P27, and P-Rb levels. As shown in (Fig.6 B), Western blot analysis revealed that knockdown of CARMA3 decreased the protein levels of Cyclin D1, CDK4, CDK6, and P-Rb but increased the levels of p27. Taken together, these results indicate that inhibition of CARMA3 expression induces cell cycle arrest in the G1 phase and suppresses lung cancer cell growth.

### Suppression of CARMA3 Inhibits p-IκB and NF-κB Activation

To determine whether CARMA3 mediates EGF-stimulated NF-κB activation in lung cancer cells, we used H1299 and A549 cells. The cells that had undergone CARMA3 siRNA treatment exhibited NF-κB inhibition in the presence or absence of EGF. Similarly, our results demonstrated that siRNA-mediated Bcl10-knockdown inhibited NF-κB luciferase expression (Fig.7 A). Our study results clearly demonstrate the essential role of the CARMA3-Bcl10-MALT1 signaling complex in EGF-induced NF-κB activation in lung cancer cells. To further verify the role of CARMA3 expression in EGF-induced NF-κB activation in cancer cells, H1299 cells and A549 cells transduced with CARMA3 siRNA were stimulated with EGF. We observed a blockade in the ability of EGF to induce p-IκB concomitant with knockdown of CARMA3, but EGF-induced IKK phosphorylation was not affected in CARMA3-deficient cells. Furthermore, the CARMA3 knockdown did not impair EGF-induced phosphorylation of ERK (Fig.7 B). Taken together, our results demonstrate that CARMA3, via IκB phosphorylation, mediates EGF-induced NF-κB activation in lung cancer cells.

## Discussion

In this study, we evaluated the expression pattern and biological role of the novel scaffold protein CARMA3 in human NSCLC. Our results demonstrated that CARMA3 protein expression in lung cancer tissues is higher compared to corresponding normal lung tissues. At the protein level, CARMA3 expression was restricted to the cytoplasm in all samples. We found a significant correlation between CARMA3 up-regulation and both TNM stage and tumor status. In addition, we demonstrated that CARMA3 up-regulation also correlated with a shorter survival rate of patients of nodal status N0 as well as EGFR status. CARMA3 has been previously demonstrated to play a pivotal role in EGFR induced activation of NF-κB [18]. EGFR mutation is significantly correlated with elevated EGFR expression, its downstream signaling, and clinical response to gefitinib therapy [21]. Thus, we further examined the relationship between CARMA3 expression and EGFR mutation. We found that the expression of CARMA3 is significantly higher in EGFR mutation cases compared to non-mutation cases. These results suggested that CARMA3 may play an important role in EGFR-mutant lung cancer. A recent report demonstrated that knockdown of several components of the NF-kB pathway specifically enhanced cell death induced by the EGFR TKI erlotinib in EGFR-mutant lung cancer cells [22]. As overexpression of CARMA3 proteins induces robust NF-kB activation [3], [4], CARMA3 and NF-kB may serve as potential companion drug targets along with EGFR in EGFR-mutant lung cancer.

CARMA3 has been reported to promote ovarian cancer cell progression [17], to increase human breast cancer cell growth and invasion [18], induce invasion of oral squamous cell carcinoma (OSCC) [23]; CARMA3 also plays an important role in atherogenesis [24] and NF-kB activation in airway epithelial cells [25]. However, the functional role remains unclear. To elucidate the biological function of CARMA3 in lung cancer, we employed siRNA to knockdown CARMA3 expression in both A549 and H1299 cell lines, which express high levels of CARMA3. We observed impaired proliferation capacity in both A549 and H1299 cells after CARMA3 knockdown. Knockdown of Bcl10, which is another component of the CBM complex, also impaired cell proliferation. Thus, CARMA3 siRNA knockdown potentially impairs malignant cell proliferation through the destruction of the CARMA3-Bcl10-MALT1 complex. Most proliferative factors influence cell growth by affecting cell cycle progression. In this study, cell cycle analyses revealed that the percentage of cells in the G1 phase was increased, whereas the percentage of cells in the S phase was decreased in CARMA3-knockdown cells compared to control cells. Knockdown of CARMA3 inhibited the G1 to S transition in cell cycle progression, which suggests the role played by CARMA3 in lung cancer cell proliferation. Cell cycle progression is modulated by a number of molecules. To determine the potential mechanism of CARMA3 concerning cell cycle regulation, we examined the effect of CARMA3 knockdown on cell cycle-related molecules. We analyzed the levels of cyclin A, cyclin D1, cyclin D3, CDK4, CDK6, p-Rb, and P27 and found that knockdown of CARMA3 decreased the protein levels of Cyclin D1, CDK4, CDK6, and P-Rb but increased the levels of p27. Cyclin D1, which drives the progression of cells into the proliferative stages of the cell cycle, plays an important role in tumorigenesis. The cyclin-D/CDK4, 6/pRB pathway has been previously demonstrated to be altered in over 80% of human tumors [26], [27]. It is a key regulator of the critical G1 to S phase transition of the cell cycle. During the G1 phase, pRB is inactivated by sequential phosphorylation events mediated by various cyclin dependant kinases (CDKs), leading to the release of the E2F transcription factors, the activation of many genes, and progression of the cell cycle [28]. The cyclin-dependent kinase inhibitor p27 (also named Kip1) is a growth inhibitory protein that blocks cell cycle progression at the G1/S transition [29]. Taken together, these results indicate that CARMA3 controls the cell cycle by regulating cyclin D1, CDK4, CDK6, p-Rb, and p27.

It has become increasingly clear that NF-κB signaling plays a critical role in cancer development and progression [30]–[34]. Our results further demonstrate a direct link between CARMA3 and NF-κB activation. The change of cell cycle progression after CARMA3 knockdown is potentially related to NF-κB. NF-κB is the subject of numerous pharmaceutical research studies as a target for anti-cancer therapy. Blocking NF-κB potentially arrests tumor cell proliferation and causes apoptosis, or enhances sensitivity to the action of anti-tumor agents. The present study demonstrated that CARMA3 knockdown inhibits NF-κB activation, thus blocking lung cancer progression and suggesting the potential importance of this finding for novel cancer therapies.

Lung cancer is the leading cause of cancer deaths in the united States and worldwide. The major form of lung cancer is NSCLC [35].Despite advances in early detection and standard treatment, NSCLC is often diagnosed at an advanced stage and has a poor prognosis. Discover valuable biomarker for the detection of NSCLC at an early stage can improve survival substantially. Now, there are many potentially useful biomarkers for lung cancer have been verified, such as CEA, CA-125, CYFRA 21-1, TPA [35]–[39]. More and more novel potential biomarkers for lung cancer have been discovered, such as SAA, DDH, EGFR, Mig-6 [40]–[43]. In this research we find CARMA3 as another potentially useful biomarker for lung cancer. Identifications of biomarkers are leading to more understanding of the molecular pathways involved in lung cancer and help to reduce the suffering and loss of life caused by the lethal disease [44].

In conclusion, our study demonstrated that CARMA3 is overexpressed in NSCLC and correlates with lung cancer progression. CARMA3 promotes lung cancer proliferation through cell cycle regulation and NF-κB regulation.

## Supporting Information

Figure S1 CARMA3 mRNA knockdown was assessed by quantitative RT-PCR.
(JPG)

Figure S2 TheCARMA3 expression in 1 normal (92#)and 17 NSCLC tissues samples(400×).
(TIF)

Figure S3 CARMA3 protein expression in NSCLC. Equal amounts of total protein (60 µg) from normal (92#)and NSCLC tissues were analyzed by Western blotting with an anti-CARMA3 antibody or an anti-β-actin antibody as loading control. Each bar represents the mean± SD of three independent experiments,compared with control(92#)Specimens used in the experiment were the same samples used in Figure S2.
(JPG)

Figure S4 Survival of N0 stage NSCLC patients correlates with the expression of CARMA3. Kaplan–Meier survival plots for patients with NSCLC and CARMA3 protein expression. Correlation between overall survival of patients with CARMA3 expression were found to be statistically significant (P = 0.042). All patients alive at their last follow-up are indicated by tick marks on the plot.
(JPG)

Table S1 Primer sequences.
(DOC)

Table S2 The expression of CARMA3 and EGFR in 16 NSCLC tissues with EGFR mutation.
(DOC)

## References

[1] Bertin J, Wang L, Guo Y, Jacobson MD, Poyet JL (2001). CARD11 and CARD14 are novel caspase recruitment domain (CARD)/membrane-associated guanylate kinase (MAGUK) family members that interact with BCL10 and activate NF-kappa B. J Biol Chem.

[2] Gaide O, Martinon F, Micheau O, Bonnet D, Thome M (2001). Carma1, a CARD-containing binding partner of Bcl10, induces Bcl10 phosphorylation and NF-kappaB activation.. FEBS Lett.

[3] Wang L, Guo Y, Huang WJ, Ke X, Poyet JL (2001). Card10 is a novel caspase recruitment domain/membrane-associated guanylate kinase family member that interacts with BCL10 and activates NF-kappa B. J Biol Chem.

[4] McAllister-Lucas LM, Inohara N, Lucas PC, Ruland J, Benito A (2001). Bimp1, a MAGUK family member linking protein kinase C activation to Bcl10-mediated NF-kappaB induction.. J Biol Chem.

[5] Grabiner BC, Blonska M, Lin PC, You Y, Wang D (2007). CARMA3 deficiency abrogates G protein-coupled receptor-induced NF-{kappa}B activation.. Genes Dev.

[6] McAllister-Lucas LM, Ruland J, Siu K, Jin X, Gu S (2007). CARMA3/Bcl10/MALT1-dependent NF-kappaB activation mediates angiotensin II-responsive inflammatory signaling in nonimmune cells.. Proc Natl Acad Sci U S A.

[7] Borthakur A, Bhattacharyya S, Alrefai WA, Tobacman JK, Ramaswamy K (2010). Platelet-activating factor-induced NF-kappaB activation and IL-8 production in intestinal epithelial cells are Bcl10-dependent.. Inflamm Bowel Dis.

[8] Marasco D, Stilo R, Sandomenico A, Monti SM, Tizzano B (2009). Generation and functional characterization of a BCL10-inhibitory peptide that represses NF-kappaB activation.. Biochem J.

[9] Hayden MS, Ghosh S (2004). Signaling to NF-kappaB.. Genes Dev.

[10] Aggarwal BB (2004). Nuclear factor-kappaB: the enemy within.. Cancer Cell.

[11] Lukiw WJ, Zhao Y, Cui JG (2008). An NF-kappaB-sensitive micro RNA-146a-mediated inflammatory circuit in Alzheimer disease and in stressed human brain cells.. J Biol Chem.

[12] Singh MV, Kapoun A, Higgins L, Kutschke W, Thurman JM (2009). Ca2+/calmodulin-dependent kinase II triggers cell membrane injury by inducing complement factor B gene expression in the mouse heart.. J Clin Invest.

[13] Panzer U, Steinmetz OM, Turner JE, Meyer-Schwesinger C, von Ruffer C (2009). Resolution of renal inflammation: a new role for NF-kappaB1 (p50) in inflammatory kidney diseases.. Am J Physiol Renal Physiol.

[14] Wang D, You Y, Lin PC, Xue L, Morris SW (2007). Bcl10 plays a critical role in NF-kappaB activation induced by G protein-coupled receptors.. Proc Natl Acad Sci U S A.

[15] Klemm S, Zimmermann S, Peschel C, Mak TW, Ruland J (2007). Bcl10 and Malt1 control lysophosphatidic acid-induced NF-kappaB activation and cytokine production.. Proc Natl Acad Sci U S A.

[16] Sun J, Lin X (2008). Beta-arrestin 2 is required for lysophosphatidic acid-induced NF-kappaB activation.. Proc Natl Acad Sci U S A.

[17] Mahanivong C, Chen HM, Yee SW, Pan ZK, Dong Z (2008). Protein kinase C alpha-CARMA3 signaling axis links Ras to NF-kappa B for lysophosphatidic acid-induced urokinase plasminogen activator expression in ovarian cancer cells.. Oncogene.

[18] Jiang T, Grabiner B, Zhu Y, Jiang C, Li H (2011). CARMA3 is crucial for EGFR-Induced activation of NF-kappaB and tumor progression.. Cancer Res.

[19] Red Brewer M, Choi SH, Alvarado D, Moravcevic K, Pozzi A (2009). The juxtamembrane region of the EGF receptor functions as an activation domain.. Mol Cell.

[20] Nicholas MK, Lukas RV, Jafri NF, Faoro L, Salgia R (2006). Epidermal growth factor receptor - mediated signal transduction in the development and therapy of gliomas.. Clin Cancer Res.

[21] Sequist LV, Bell DW, Lynch TJ, Haber DA (2007). Molecular predictors of response to epidermal growth factor receptor antagonists in non-small-cell lung cancer.. J Clin Oncol.

[22] Bivona TG, Hieronymus H, Parker J, Chang K, Taron M (2011). FAS and NF-kappaB signalling modulate dependence of lung cancers on mutant EGFR.. Nature.

[23] Rehman AO, Wang CY (2009). CXCL12/SDF-1 alpha activates NF-kappaB and promotes oral cancer invasion through the Carma3/Bcl10/Malt1 complex.. Int J Oral Sci.

[24] McAllister-Lucas LM, Jin X, Gu S, Siu K, McDonnell S (2010). The CARMA3-Bcl10-MALT1 signalosome promotes angiotensin II-dependent vascular inflammation and atherogenesis.. J Biol Chem.

[25] Medoff BD, Landry AL, Wittbold KA, Sandall BP, Derby MC (2009). CARMA3 mediates lysophosphatidic acid-stimulated cytokine secretion by bronchial epithelial cells.. Am J Respir Cell Mol Biol.

[26] Vogelstein B, Kinzler KW (2004). Cancer genes and the pathways they control.. Nat Med.

[27] Nevins JR (2001). The Rb/E2F pathway and cancer.. Hum Mol Genet.

[28] Giacinti C, Giordano A (2006). RB and cell cycle progression.. Oncogene.

[29] Chu IM, Hengst L, Slingerland JM (2008). The Cdk inhibitor p27 in human cancer: prognostic potential and relevance to anticancer therapy.. Nat Rev Cancer.

[30] Wichlinski L (1969). [New solvent system for thin-layer chromatography of ergot alkaloids].. Acta Pol Pharm.

[31] Basseres DS, Baldwin AS (2006). Nuclear factor-kappaB and inhibitor of kappaB kinase pathways in oncogenic initiation and progression.. Oncogene.

[32] Karin M (2006). Nuclear factor-kappaB in cancer development and progression.. Nature.

[33] Mantovani A, Allavena P, Sica A, Balkwill F (2008). Cancer-related inflammation.. Nature.

[34] Prasad S, Ravindran J, Aggarwal BB (2010). NF-kappaB and cancer: how intimate is this relationship.. Mol Cell Biochem.

[35] Siegel R, Naishadham D, Jemal A (2012). Cancer statistics, 2012.. CA Cancer J Clin.

[36] Barlesi F, Gimenez C, Torre JP, Doddoli C, Mancini J (2004). Prognostic value of combination of Cyfra 21–1, CEA and NSE in patients with advanced non-small cell lung cancer.. Respir Med.

[37] Pujol JL, Molinier O, Ebert W, Daures JP, Barlesi F (2004). CYFRA 21–1 is a prognostic determinant in non-small-cell lung cancer: results of a meta-analysis in 2063 patients.. Br J Cancer.

[38] Ando S, Kimura H, Iwai N, Yamamoto N, Iida T (2003). Positive reactions for both Cyfra21–1 and CA125 indicate worst prognosis in non-small cell lung cancer.. Anticancer Res.

[39] Barak V, Goike H, Panaretakis KW, Einarsson R (2004). Clinical utility of cytokeratins as tumor markers.. Clin Biochem.

[40] Cho WC, Yip TT, Cheng WW, Au JS (2010). Serum amyloid A is elevated in the serum of lung cancer patients with poor prognosis.. Br J Cancer.

[41] Huang LJ, Chen SX, Huang Y, Luo WJ, Jiang HH (2006). Proteomics-based identification of secreted protein dihydrodiol dehydrogenase as a novel serum markers of non-small cell lung cancer.. Lung Cancer.

[42] Nishio K, Arao T, Shimoyama T, Fujiwara Y, Tamura T (2005). Translational studies for target-based drugs.. Cancer Chemother Pharmacol.

[43] Li Z, Dong Q, Wang Y, Qu L, Qiu X (2011). Downregulation of Mig-6 in nonsmall-cell lung cancer is associated with EGFR signaling..

[44] Cho WC (2007). Potentially useful biomarkers for the diagnosis, treatment and prognosis of lung cancer.. Biomed Pharmacother.

