# Supplementary figures and images for: Overexpression of CARMA3 in Non-Small-Cell Lung Cancer Is Linked for Tumor Progression
Source: PLoS One. 2012 May 15;7(5):e36903. doi: 10.1371/journal.pone.0036903 (PMC3352848; doi:10.1371/journal.pone.0036903)

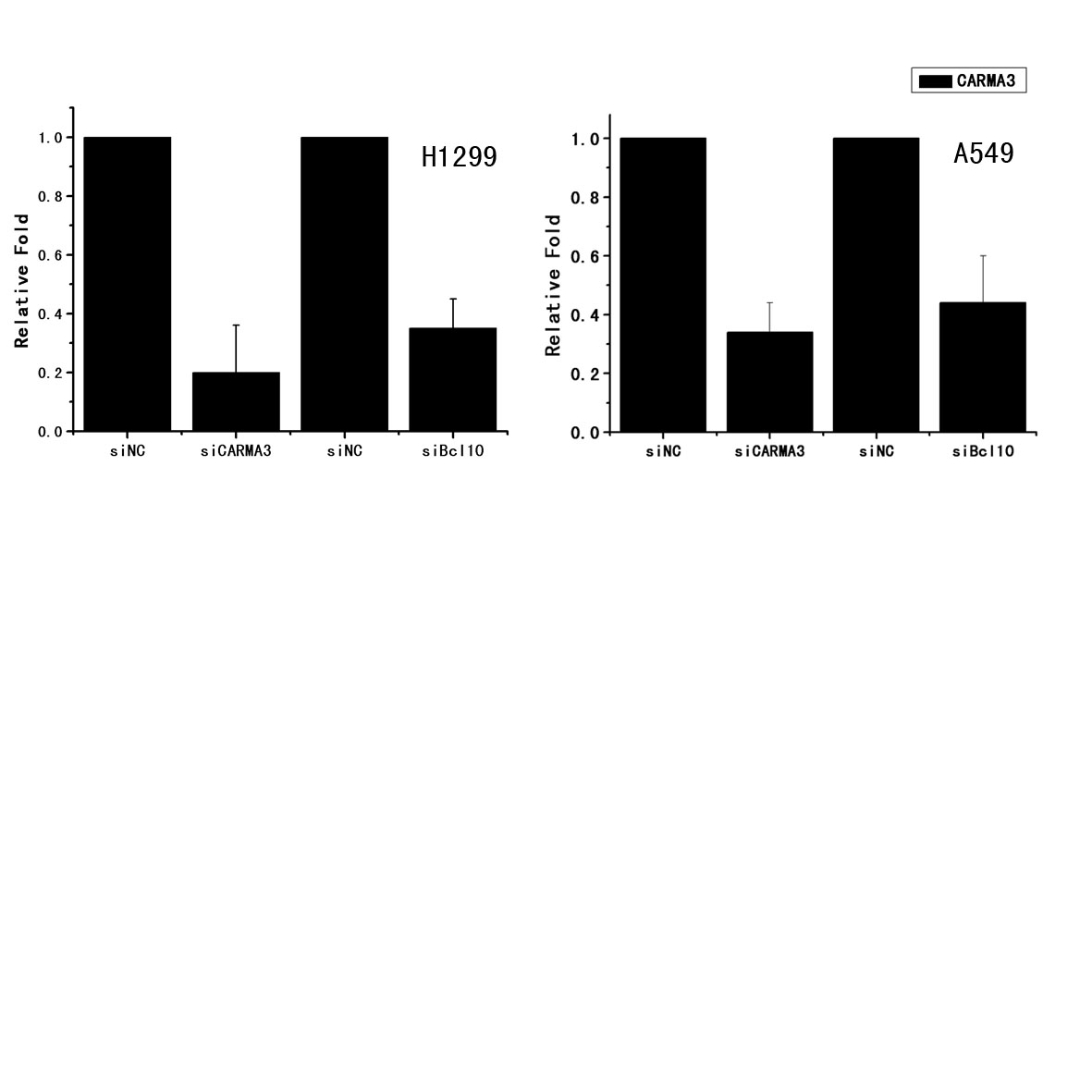

Supplement: Figure S1 — CARMA3 mRNA knockdown was assessed by quantitative RT-PCR. (JPG) [file pone.0036903.s001.jpg]

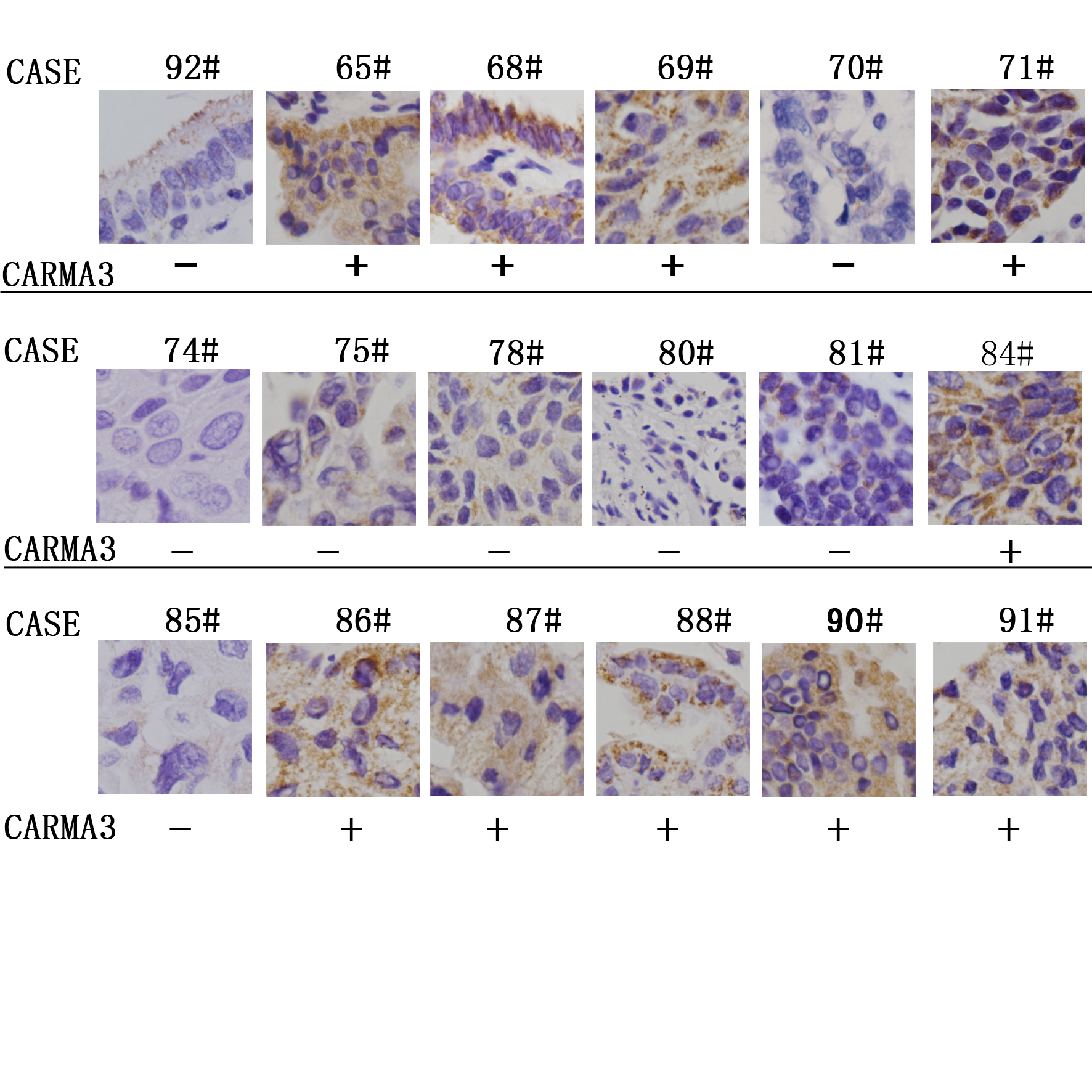

Supplement: Figure S2 — TheCARMA3 expression in 1 normal (92#)and 17 NSCLC tissues samples(400×). (TIF) [file pone.0036903.s002.tif]

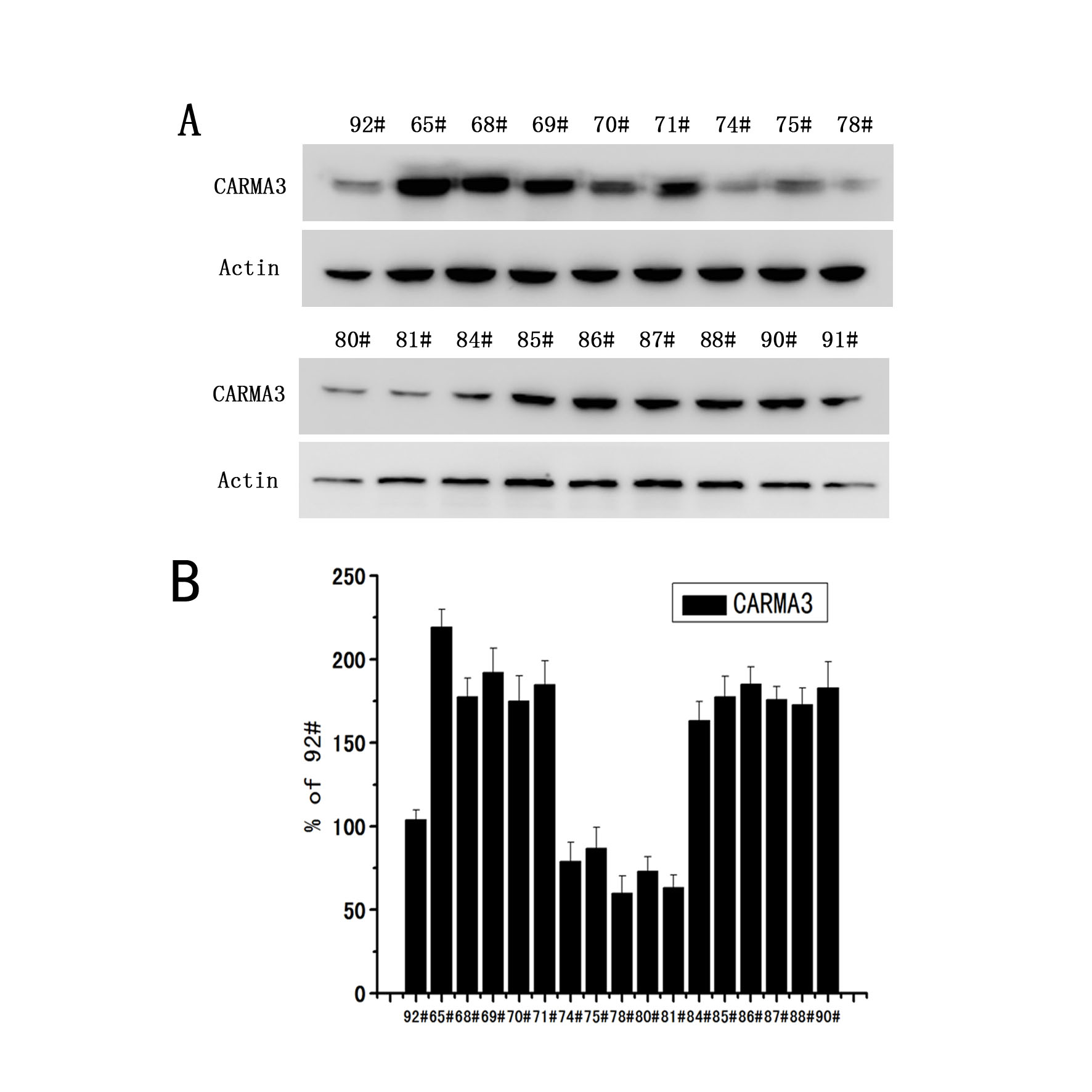

Supplement: Figure S3 — CARMA3 protein expression in NSCLC. Equal amounts of total protein (60 µg) from normal (92#)and NSCLC tissues were analyzed by Western blotting with an anti-CARMA3 antibody or an anti-β-actin antibody as loading control. Each bar represents the mean± SD of three independent experiments,compared with control(92#)Specimens used in the experiment were the same samples used in Figure S2. (JPG) [file pone.0036903.s003.jpg]

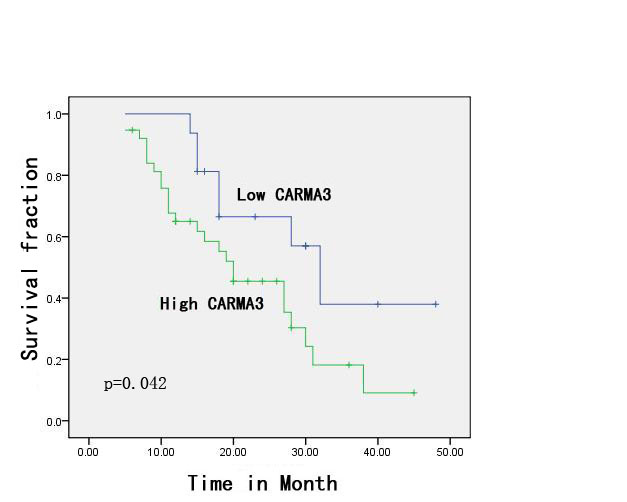

Supplement: Figure S4 — Survival of N0 stage NSCLC patients correlates with the expression of CARMA3. Kaplan–Meier survival plots for patients with NSCLC and CARMA3 protein expression. Correlation between overall survival of patients with CARMA3 expression were found to be statistically significant (P = 0.042). All patients alive at their last follow-up are indicated by tick marks on the plot. (JPG) [file pone.0036903.s004.jpg]
