# Supplementary material for: Overexpression of CARMA3 in Non-Small-Cell Lung Cancer Is Linked for Tumor Progression
Source: PLoS One. 2012 May 15;7(5):e36903. doi: 10.1371/journal.pone.0036903 (PMC3352848; doi:10.1371/journal.pone.0036903)
Supplement: Table S1 — Primer sequences. (DOC) [file pone.0036903.s005.doc]

| Supplementary table 1. Primer sequences | |
| --- | --- |
| Name | Primer sequences |
| ACTIN forward | 5’-ATAGCACAGCCTGGATAGCAACGTAC-3’ |
| ACTIN reverse | 5’-CACCTTCTACAATGAGCTGCGTGTG-3’ |
| CARMA3 forward | 5’-TCTTCCACCGTTGCCAATCT-3’ |
| CARMA3 reverse | 5’-TTCGCCTGCCAGGAACATC-3’ |
| Bcl10 forward | 5’-GGAGAACTGCCAGAAACTGACC-3’ |
| Bcl10 reverse | 5’-GCCTACAGCACACTGGTTG-3’ |
