# Supplementary material for: Overexpression of CARMA3 in Non-Small-Cell Lung Cancer Is Linked for Tumor Progression
Source: PLoS One. 2012 May 15;7(5):e36903. doi: 10.1371/journal.pone.0036903 (PMC3352848; doi:10.1371/journal.pone.0036903)
Supplement: Table S2 — The expression of CARMA3 and EGFR in 16 NSCLC tissues with EGFR mutation. (DOC) [file pone.0036903.s006.doc]

| Supplementary table2.The expression of CARMA3 and EGFR in 16 NSCLC tissues with EGFR mutation | | | | | |
| --- | --- | --- | --- | --- | --- |
| Patient | Gender | Age | EGFR Mutation | CARMA3 status | EGFR status |
| 1 | Female | 75 | Del19 | ＋ | ＋ |
| 2 | Male | 72 | Del19 | ＋ | ＋ |
| 3 | Female | 69 | L858R | ＋ | ＋ |
| 4 | Male | 72 | L858R | ＋ | ＋ |
| 5 | Female | 49 | Del19 | ＋ | ＋ |
| 6 | Female | 47 | L858R | ＋ | ＋ |
| 7 | Male | 71 | Del19 | ＋ | ＋ |
| 8 | Male | 82 | L858R | ＋ | ＋ |
| 9 | Female | 40 | Del19 | － | ＋ |
| 10 | Male | 49 | L858R | ＋ | － |
| 11 | Female | 65 | Del19 | ＋ | ＋ |
| 12 | Male | 67 | L858R | ＋ | ＋ |
| 13 | Female | 42 | Del19 | ＋ | ＋ |
| 14 | Female | 63 | L858R | － | ＋ |
| 15 | Male | 47 | L858R | ＋ | ＋ |
| 16 | Male | 74 | L858R | ＋ | ＋ |

Abbreviations: Mig-6, mitogen-inducible gene-6; EGFR,epidermal growth factor receptor; NSCLC, non-small-cell lung cancer; Del19，deletion mutation in exon 19 of the EGFR gene；L858R ，All L858R mutations are 2573T>G.
